# Transition from Paediatric to adult health services: Aspirations and practices of human flourishing

**DOI:** 10.1080/17482631.2023.2278904

**Published:** 2023-11-23

**Authors:** Nicole Padley, Dina Moubayed, Amélie Lanteigne, François Ouimet, Marie-José Clermont, Anne Fournier, Eric Racine

**Affiliations:** a Pragmatic Health Ethics Research Unit, Institut de recherches cliniques de Montréal, Montréal, QC, Canada; b Département de pédiatrie (section médecine de l'adolescence), Centre Hospitalier Universitaire Sainte-Justine, Montréal, Québec, Canada; c Département de médecine, Université de Montréal, Montréal, QC, Canada; d Département de pédiatrie, CHU Sainte-Justine, Montréal, Québec, Canada; e Département de pédiatrie, Université de Montréal, Montréal, QC, Canada; f Département de médecine sociale et préventive, École de santé publique de l’Université de Montréal, Montréal, QC, Canada; g Department of Medicine (Division of Experimental Medicine), McGill University, Montréal, QC, Canada; h Department of Neurology and Neurosurgery, McGill University, 3801 Rue University, Montréal, QC, Canada

**Keywords:** Transition, autonomy, paediatrics, ethics, human flourishing

## Abstract

**Background:**

Transition from paediatric to adult care is challenging for youths with a chronic condition. Most transition programmes place high value in autonomy and independence. We undertook a qualitative study to: (1) identify the needs and aspirations of youths and (2) better understand the well-being and flourishing of youths.

**Methods:**

Semi-structured interviews were conducted with youths, parents of youths and healthcare professionals recruited from four clinics. Thematic analysis focused on: (1) perceptions of transition; (2) key aspects of human flourishing during transition; and (3) salient concerns with respect to the transition and dimensions of human flourishing.

**Results:**

54 interviews were conducted. Perceptions of transition clustered around: (1) apprehension about adult care; (2) lack of clarity about the transition process; (3) emotional attachment to paediatric healthcare professionals; (4) the significance of the coinciding transition into adulthood. Fourteen salient concerns (e.g., Knowledge and information about the transition, Parental involvement in healthcare) were identified with corresponding recommendations. Salient concerns related to important dimensions of human flourishing (e.g., environmental mastery, autonomy).

**Discussion and conclusion:**

The flourishing of youths is affected by suboptimal transition practices. We discuss the implications of our findings for environmental mastery, contextual autonomy, and the holistic and humanistic aspects of transition.

## 1 Introduction

Transition from paediatric to adult care is both a crucial and challenging moment in the lives of adolescents and young adults (youths) living with chronic physical conditions as well as mental (Anderson et al., 2022) and neurodevelopmental conditions (Racine et al., 2014; Swift et al., 2013). While youths may experience unique obstacles depending on the nature of their condition, they may also face significant challenges that are associated with suboptimal transition practices such as cessation of medication, important delays for adult care admission, and discontinuation of healthcare (Toulany et al., 2022). Youth often require support to deal effectively with new systems of care and adaptation to the many co-occurring changes at the time of early adulthood (e.g., housing, work, higher education) while some need ongoing support from parents and caregivers into adulthood (Toulany et al., 2022). Although significant attention is now given to the transition, most programmes implemented in hospitals focus on the “transfer of care”, namely, the transfer of medical records from one system to another (Betz et al., 2016; Clemente et al., 2017; Lanteigne et al., 2021). Additionally, transition programmes are generally oriented towards maximizing medical compliance and treatment adherence, thereby placing high value in concepts such as autonomy and independence while more relational and less performance-oriented goals may be cherished by some youths who cannot realistically attain full independence (Gibson et al., 2014; Hamdani et al., 2015; Larivière-Bastien et al., 2013; Racine et al., 2014). This raises questions about who truly benefits from the goals and outcomes of transition programmes. Autonomy-centred transition programmes may not reflect the values of all youths and their families, whose set of values may differ from those embedded in the healthcare system. Ensuring that transition programmes are conceived *for* and *by* youths and their families through participatory research could help minimize this pitfall. Developing transition programmes for youths means ensuring these programmes aim to maximize their well-being, rather than fostering a narrow conception of autonomy or independence. Participatory research on paediatric-adult healthcare transition is not well developed such that its merits need to be better ascertained, but in health research more broadly, participatory research helps tailor services to the needs of patients, empower patients in the participatory research process, and increase sensitivity of professional practices to the needs of patients and the recognition of their personhood and agency (Jagosh et al., 2012). Involving youths and their families in the research process and implementation of transition programmes is thus a promising approach to integrate their values, concerns, and visions of well-being into the design and conceptions of these programmes given the general benefits of participatory research.

In this context, the concept of “human flourishing” offers a promising outlook on healthcare transition research. As a conceptualization of well-being, flourishing has the advantage of accounting for the role of meaning-making narratives in the pursuit of one’s own life objectives (Lanteigne et al., 2021). Human flourishing is therefore more encompassing than competing concepts such as quality of life, happiness or satisfaction as it proposes that a meaningful and flourishing life is defined by the person themself. Ryff and Singer (Ryff & Singer, 2008) have proposed a six-dimensional model of flourishing^1^ that integrates concepts such as “self-acceptance”, “personal growth”, “purpose in life” and “positive relation with others”, together with “autonomy” and “environmental mastery” to create a holistic perspective of well-being that merges insights from different schools of thought in psychology and philosophy (see online-only table: Key dimensions of human flourishing). Flourishing has proven to be a powerful predictor of physical and psychological health (VanderWeele, 2017). For example, research in adults shows that key dimensions of Ryff’s model are correlated with longevity as well as diminished morbidity through various physiological mechanisms (Friedman & Ryff, 2012; Kim et al., 2022; Ryff & Singer, 1998; Schaefer et al., 2013; Urry et al., 2004) such as specific gene expression, physiological regulation and regulation of emotion. More importantly, flourishing may offer a conceptual lens to understand the lived experience of youths and their families during healthcare transition, and consequently ensure that transition programmes are built in a way that maximizes their well-being. A recent literature review has highlighted that very few studies evaluate healthcare transition programmes using flourishing as a guiding concept (Lanteigne et al., 2021). This study is the first phase of a four-phase participatory project that ultimately aims to implement new interventions for the healthcare transition in a paediatric care hospital in Montreal, Canada. It is inspired by pragmatist theory which stresses the active role of people as agents in their lives, including their moral lives (Aiguier & Cobbaut, 2016; Aiguier & Loute, 2016) and that accordingly, participatory approaches are ideally suited to empower and capacitate (Clark et al., 2019; Zimmermann, 2006). The first phase reported in this paper follow the line of more conventional qualitative research and is part of a broader participatory study.^2^

## 2 Methods

### 2.1 Study aims

We undertook qualitative research inspired by pragmatist theory and embedded in a broader participatory research project to pursue the following aims:

1. Identify the needs and aspirations of youths, parents, and healthcare professionals involved in the transition from paediatric to adult care and;
2. Better understand the well-being and flourishing of youths in the context of the transition of care.

The qualitative research design adopted implies that youths, parents, and healthcare professionals contribute to understanding aspirations for the transition but also take part in envisioning and enacting solutions. A series of individual, semi-structured interviews were conducted to explore the perspectives of the parties involved in transition care. Subsequent steps of the broader participatory project will build upon these data to help tailor healthcare transition programmes based on the active engagement of youths, parents, and healthcare professionals. A subsequent survey will be developed to validate the aspirations and needs of youths and parents based on the results of the interviews and discussions with the advisory committee composed of parents, youths, and healthcare professionals. Based on this survey, and the needs expressed, participatory interventions will be codeveloped with stakeholders, implemented, and evaluated. Overall, the broader study aims to support the further development of an institutional transition programme and to tailor this programme to the needs and aspirations of youths and parents.

### 2.2 Ethics approval

Ethics approval from the Research Ethics committee of the Centre hospitalier universitaire Sainte-Justine (CHU-Saint-Justine), approval no. 2019–2133] and of the Institut de recherches cliniques de Montréal (IRCM) was sought and granted (approval no. 2020–1064).

### 2.3 Participants

Interview participants consisted of three groups: 1) youths between 15 and 25 years old living with a chronic condition who were approaching or had recently experienced transition of care, 2) parents of youths between 15 and 25 years old living with a chronic condition who were approaching or had recently experienced transition of care, 3) healthcare professionals working at a paediatric care centre in Montreal, Quebec with experience assisting youths and their families during their transition to adult care. Parents participating in the study were not required to have a child participating in the study as well. Healthcare professionals included physicians, nurses, social workers, and psychologists working with youths from the studied clinics. The age range of youth participants was determined based on practical and methodological reasons (e.g., topic of transition was understandable and relevant for pre-transition youth; self-identification to youth and not-so-distant experiences for post-transition youth) as the age of transition is typically 18 in the setting studied.

### 2.4 Recruitment procedure

Interview participants were recruited at The Centre hospitalier universitaire Sainte-Justine (CHU Sainte-Justine) through a clinical research coordinator affiliated to the institution, from four clinics who accepted to participate in the research project: cardiology, nephrology, gastroenterology and adolescent medicine. The first three clinics centre on physical chronic illnesses while the fourth covers psychiatric/mental health conditions as well. The clinical research coordinator explained the study, answered questions and obtained written consent from those who agreed to participate. Consent included the agreement to be interviewed, to be audio-recorded, to receive compensation and to have interview data published anonymously. Consent was obtained with approval from a legally authorized representative for participants under 18 years old. Participants chose their interview date and time in collaboration with the research team. Interview participants were offered financial compensation of 50$ for their participation.

### 2.5 Interview process

Individual interviews—initially planned as in-person focus groups which were not possible due to the COVID-19 pandemic—were conducted via Zoom and audio-recorded. The evening prior to the interview, the researcher conducting the interview shared a password-protected Zoom link with the participant. Audio-recorded French-language interviews began in August 2020 and were conducted over the course of the following 12 months on a rolling basis as the clinical research coordinator recruited participants. Interviews generally lasted between 30 minutes to one hour. Interview recordings were sent to a professional transcription service for verbatim transcription.

### 2.6 Interview guide

An initial interview guide was devised based on the study objectives. A series of pre-test interviews were conducted on a subsample of participants. The pre-test sample (n = 9) consisted of 3 youths (1 cardiology, 1 nephrology, 1 gastroenterology), 3 parents (1 cardiology, 2 nephrology) and 3 healthcare professionals (1 cardiology, 1 nephrology, 1 gastroenterology). This allowed researchers to test the comprehensibility of questions, receive feedback from participants, and refine the interview guide accordingly. Pre-test interviews were not analysed as data and were not included in the study sample.

The final interview guide was then devised, and interviews conducted. The interview began with a preamble about the context and objectives of the study. It was divided into three sections: 1) perceptions of the healthcare transitions and an ideal transition programme; 2) flourishing in the context of the transition; and 3) ethical matters related to healthcare and the healthcare transition. Throughout the interview, researchers could ask follow-up questions to probe participants to further elaborate on their perspectives. Participants were invited to share any additional concerns or subjects at the end of the interview. Overall, the semi-structured nature of the interview and the broadness of the questions allowed participants to share their views freely in a conversational manner to explore a broad scope of issues related to the transition according to participants’ perspectives and experiences.

### 2.7 Coding and data analysis

Coding of interviews followed thematic analysis (Braun & Clarke, 2006) and relied on both deductive and inductive coding strategies. An initial coding guide was piloted on a sub-sample of interviews by one researcher (AL) who was familiar with the data through conducting interviews. This initial coding structure was then refined and further elaborated through several coding tests and team meetings to yield the final coding structure. Three areas of content were retained, each sub-divided in sub-themes: (1) perceptions of transition based on interviewee’s responses to their understanding and attitude towards transition (study aim 1); (2) key aspects of human flourishing relevant to healthcare transition, which pulled from the explicit discussion on dimensions of flourishing as well as the discussion of ethical issues in healthcare transitions (study aims 1 and 2); and (3) salient concerns identified with respect to the transition and different dimensions of human flourishing (study aim 2). These concerns were pulled from the discussion of ethical matters as well as all issues and challenges reported. They were further broken down into (a) statements affirming the concern, (b) reported relevant challenges and (c) relevant recommendations related to the concern. Coding for areas of content 1 and 3 was mostly inductive and sub-themes were derived from initial open coding and iterative refinement to generate the final sub-themes that were drawn from the issues and topics frequently raised by interviewees. Coding for area 2 was deductive and relied on dimensions of human flourishing found in Ryff and Singer (2008)’s integrative six-dimension model of flourishing. Interview segments were coded with one or more dimensions of human flourishing when these were explicitly invoked or where the coder interpreted the dimension(s) to be relevant based on the definitions proposed in Ryff and Singer’s model. Coding was supported by MaxQDA and undertaken by three of the authors. Throughout the coding, sub-samples of interviews were exchanged between coders to support inter-coder consistency in the application of codes. Instances of disagreement between coders were recorded and presented to the senior author (ER) for discussion and arbitration.

### 2.8 Data analysis and presentation

Basic participant information is presented in section 1. Section 2 provides a vision of participants’ general perceptions of the transition. Relevant thematic content for this section is synthesized and presented narratively and supported by illustrative citations. Content for the theme of salient concerns with respect to the transition is presented in a separate table and organized thematically (Table I). The dimensions of flourishing (section 3) are reported in order of significance, determined by the saliency of these dimensions amongst participants and their prevalence in the coding (See online-only Table: Key dimensions of human flourishing). Relevant thematic content for this third section is also synthesized and presented narratively and supported by illustrative citations. It is also divided into sub-themes given its complexity. Furthermore, linkages between salient concerns (Table I) and dimensions of human flourishing are noted. Translation of cited excerpts of interviews was undertaken by a fully bilingual team member (FO) and validated by another bilingual member (ER). For readability purposes, we provide quick and identifiable reminder definitions of key aspects of human flourishing (in section 3 of the results) within the results although these are not results *per se*. In the preparation of the manuscript, we also consulted youths and parents (*N* = 3) who were invited to comment on the manuscript for readability and relevance. Participants are anonymized. Their clinical affiliation (cardiology, nephrology, gastroenterology, adolescent medicine is identified as C, N, G, AM) and their status as Y, P, HCP for youths, parents and healthcare professionals, and their identify numbered (e.g., C-Y1, AM-HCP2)

**Table I.** Salient concerns regarding transition.

Salient concerns	Participants’ affirmations of the concern	Challenges associated with the concern	Proposed recommendations regarding the concern
1. Continuity in care	Participants affirmed that the principal objective of the transition is the continuation of the patient’s care—follow-ups and treatments and care service—in the adult centre. Participants affirmed that the transition should be a fluid and gradual process. Participants emphasized the importance of communication and coordination between paediatric and adult care centres. Participants emphasized the importance of guidance and accompaniment throughout the transition. Participants emphasized the importance of preparation and coordination in advance.	Participants expressed stress and instability towards the transition and perceived the transition as an abrupt cut-off from care. Parents and youths lacked reassurance about the transfer of their care to an adult care provider and adult care centre. Parents and youths recounted experiences where youths struggled to access services and receive care during the transition. They described a gap between paediatric care and adult care. Participants identified a lack of coordination of the transition process and a lack of communication between paediatric and adult care.	A pivotal resource-person, such as a transition coordinator, to offer guidance throughout the transition. A pre-transition meeting with the patient, their parent(s), the paediatric care provider and the adult care provider doctor to facilitate communication between care centres. Follow-up by the paediatric doctor to ensure the patient has successfully transferred. Preparation and coordination of the adult care provider in advance.
2.Multidisciplinary care	Participants affirmed that paediatric care facilitated access to diverse care services as these services were congregated and coordinated in one care centre. Participants affirmed the importance of psychosocial resources in particular.	Participants worried that the transition would interrupt patients’ access to diverse care services. Parents and youths anticipated difficulty accessing services externally due to the organization of the adult healthcare system. Participants expressed particular concern about the loss of psychosocial support youths had received in paediatric care.	A pivotal resource-person who coordinates diverse care services. Integration of the entire care team into the transition. Coordination and communication between the diverse care disciplines.
3. Adapting to a new care system	Participants affirmed the importance of familiarizing with the new care centre and the new care team. Youths anticipated a period of adaptation to the new care centre, the adult care system, and the different care practices. Some youths who had undergone a transition to adult care reported that they adapted to the new centre and system.	Parents and youths worried about the quality of care, frequency of follow-ups, and organization of care in the adult centre. Parents and youths anticipated greater difficulty accessing resources in adult care due to the organization of the adult care system. Some parents and youths anticipated difficulty adapting to these differences. Some parents and youths expressed uncertainty and perceived adult care as unknown.	Pre-transition visit to the adult care centre. Familiarization with the adult care establishment and healthcare professionals. Transition coordinator to guide youths in their first visit(s) in adult care. Informational guide about the new care centre, the adult healthcare system and references to resources.
4. Knowledge and information about the transition	Participants affirmed the importance of equipping parents and youths with information on the transition to reduce uncertainty towards the process. Parents and youths affirmed that information on the transition process would reduce stress and help them anticipate the transition. Parents and youths wanted more information on the details on their transition in particular—i.e., when, to where and to whom they would be transferred. Parents and youths valued the opportunity to ask questions about the transition process.	Parents and youths expressed uncertainty about the transition progress. Parents and youths lacked knowledge about the details of their transition—i.e., when, to where and to whom they would be transferred. Parents and youths experienced stress and anxiety anticipating the transition without information. Parents and youths wanted to be informed earlier and had not been informed about the transition until the last minute.	Informational resources: pamphlet or videos on the transition process. Pediatric healthcare professionals: pre-transition explanation and discussion of the transition. Participants identified 16–17 years old as the ideal time to open this discussion with patients. Pediatric care centre: offer a group training and question-session on the transition.
5. Knowledge and information about the medical condition	Participants affirmed the importance of patients’ knowledge of their medical condition: diagnosis, pathology, treatment history, implications, and consequences. Youths emphasized the importance of this knowledge for their ability to explain their condition to others. Youths felt knowledge was reassuring and prepared them for the transition. Youths, parents, and health care professionals reported that explaining and quizzing youths on their medical condition is a good practice in paediatric care.	Youths reported difficulty accessing reliable sources of information on their medical condition. Healthcare professionals reported that youths widely lack knowledge of their medical condition.	Pediatric healthcare professionals: provide an in-depth explanation of youths’ medical condition. Evaluate patients’ knowledge before the transition. Website containing information on the youth’s medical condition. A notebook or pamphlet recording the details of the patient’s medical condition and history.
6. Patient involvement in healthcare decisions	Participants affirmed the importance of youths’ participation in discussions and decisions surrounding their health and healthcare plan. Parents and youths emphasized the importance of making informed decisions in light of information on their medical condition and its implications. Youths desired greater consideration of their perspectives from healthcare professionals.	Youths were not addressed or consulted on health-related issues, particularly in paediatric care. Some youths felt insecure making decisions without their parents and worried about making decisions alone in adult-centred care. Some parents were concerned about their child’s ability to make their own decisions.	Healthcare professionals should promote active patient involvement in clinical practice. Offer and explain choices to patients; consult patients directly and listen to their perspectives on their care. Healthcare professionals should equip youths with medical knowledge and information.
7. Health management	Participants identified specific tasks that are important for maintain the youth’s health: Organizing prescriptions and medications; Arranging medical appointments; Attending medical appointments alone Possessing knowledge of their medical condition and history; Monitoring and responding to symptoms.	Some youths reported difficulty managing their healthcare affairs and required help from their parents. Youths and healthcare professionals reported reliance on parents as an obstacle to the development of health management abilities. Healthcare professionals reported that many youths had not developed health management skills by the time of the transition.	Preparatory training and education on health management throughout paediatric care. Preparatory educational and informational resources that promote health management. Parents gradually transfer responsibility and support the development of health management skills throughout adolescence.
	Participants noted that these skills should develop progressively, starting in early adolescence. Healthcare professionals and youths considered it important for youths to develop the abilities to take charge of their own health and healthcare independently. Parents discussed their role in developing their child’s health management skills throughout adolescence. Some parents were confident in their child’s ability to manage their own health and healthcare affairs. Some youths were confident in their ability to take charge of their own health and healthcare affairs.		
8. Variability in autonomy	Participants noted that youths develop and mature at varying rates, resulting in varying capacities for autonomy and independence. Participants noted that youths may not have the capacity for complete independence. A youth may be capable of assuming some responsibilities (e.g., taking their medication) but not others (e.g., making their own decisions).	Participants were concerned that youths might not be able to meet the level of autonomy expected of them in the adult care system. Parents were concerned that some youths were not apt to be independent and responsible. Participants noted that cognitive delays and mental illness may inhibit youths’ capacity for autonomy.	Pre-transition evaluation of youths’ maturity and readiness for independence. Adapting resources and support to youths’ needs and capacities. Recognition of the parental role and parental support in adult-centred care.
9. Parental involvement in healthcare	Participants emphasized the high level of involvement of parents in paediatric care. They described parents’ support and maintenance of their child’s health and healthcare affairs. Youths and parents expressed reassurance and security in parental presence and support. Some parents and youths anticipated ongoing parental accompaniment in the adult healthcare system. Some youths were comfortable with greater independence from their parents. They generally felt this independence was respected by their parents. Some parents made efforts to promote their child’s independence and health management skills.	Healthcare professionals reported dependence on parents as an obstacle to youths’ independence and readiness for the transition. Some parents and youths expressed resistance and stress towards the expectation of decreased parental involvement and accompaniment in adult-centred care. Some parents were not comfortable diminishing their involvement. Some youths were not comfortable with assuming independence. Both were due to insecurity in the youth’s ability to maintain their health.	Pediatric healthcare professionals should consult patients on the degree to which they want their parents involved and present in their appointments. Parents should begin teaching their child health management skills throughout adolescence and support their gradual independence.
10. Confidentiality	Participants discussed confidentiality in three respects: 1. Patients’ right to privacy in medical consultations. 2. Patients’ right to authorize and refuse care. 3. Permission to share patients’ information. Participants felt that the transition would pose issues for confidentiality as these rights preceded the transition. Youths felt their confidentiality was respected by parents throughout paediatric care. Youths felt comfortable expressing their needs for privacy to their parents. Youths appreciated when healthcare professionals proposed the option to consult without their parents present. Parents accepted and respected their child’s rights to privacy and confidentiality. Parents were at ease when they trusted their child to communicate important information. Healthcare professionals emphasized the importance of educating youths on their rights to confidentiality.	Parents worried about their child’s ability to conduct medical consultations without their supervision. Some parents did not accept the youth’s right to keep information private. Parents considered it more important to be informed.	Pediatric healthcare professionals should systematically offer patients the option to consult their healthcare provider without parents present. Healthcare professionals can help promote confidentiality by asking parents to step outside if the patient requests privacy. Pediatric healthcare professionals should educate adolescent patients on their rights to confidentiality.
11. Relational continuity with healthcare professionals	Parents and youths emphasized the importance of establishing trust in their new healthcare professionals. Parents and youths described a strong affective bond with their paediatric healthcare team. Parents and youths emphasized the importance of healthcare professionals’ knowledge of the patient. They valued their paediatric healthcare team’s knowledge of the patient and their history. Parents and youths valued the opportunity to meet their new healthcare team before the transition. Healthcare professionals recognized the close ties between youths/parents and healthcare professionals	Parents and youths expressed stress and worry towards the transition due to the fact of leaving their paediatric healthcare professionals. Parents and youths perceived the new healthcare professionals as unfamiliar, less caring, or less empathetic. Parents and youths perceived the transition as a loss of the quality care from paediatric healthcare professionals. Healthcare professionals emphasized the affective attachment between patients and paediatric healthcare professionals as a major obstacle of the transition.	Pre-transition meeting between patients, parents, paediatric healthcare professionals and new healthcare professionals to foster familiarization and the establishment of trust. Referral from paediatric healthcare professionals to new healthcare professionals to augment trust. Collaboration between the paediatric and new healthcare professionals to transfer knowledge on the patient.
12. Social connection	Youths and parents affirmed the importance of ensuring youths do not feel alone or isolated. Youths valued a sense of belonging in their relationships with others. Parents described their role in reducing their child’s isolation by offering support. Youths particularly valued the opportunity to exchange with others with similar medical conditions as they shared similar life experiences and difficulties. Youths discussed the importance of their social network’s (friends and family) understanding of their experience with a chronic illness. Healthcare professionals affirmed the importance of social connection for youths’ well-being.	Parents worried about youths that lacked parental support. Parents and youths recognized that living with a chronic illness could create insecurity and social isolation. Youths expressed that their chronic illness can set them apart from others. Youths expressed difficulty being understood by others. Youths were not connected with other youths with similar illnesses. Healthcare professionals recognized isolation and lack of network for youths with chronic illnesses.	Psychologists and social workers can provide support for youths. Support groups with other youths with chronic illnesses to share experiences.
13. Navigating life with chronic illness	Youths and parents reported that their medical condition impacts other spheres of their life notably their recreational, academic and vocational aspirations. Youths considered it important to develop awareness and acceptance of the implications of their chronic illness. Parents offered emotional support and helped their child cope with their chronic illness. Parents and youths affirmed that information on the medical condition allowed youths to anticipate obstacles and make informed life choices. Youths valued encouragement from their parents and healthcare professionals, rather than focusing on limitations.	Participants reported that youths may experience discouragement due to the limitations posed by their chronic illness. Participants explained that youths may feel set apart from their peers due to their chronic illness. Youths were troubled by the lack of consideration of their broader life spheres from healthcare professionals. Youths reported that a lack of medical information inhibited them from aligning their aspirations and interests with their chronic illness.	Healthcare professionals should ask about patients’ future aspirations. Healthcare professionals should advise patients of the broader implications of their chronic illness. Healthcare professionals should help youths make choices about their recreational, educational, and vocational pursuits. Psychosocial support would help with coping and emotional distress.
14. Development into adulthood	Participants noted that the medical transition overlapped with a period of personal evolution and development. They highlighted the importance of youths’ development beyond the medical scope. Participants identified the following as important elements of youths’ lives: education and schooling; interests and recreational activities; job & career choices; socialization; and independence and responsibility. Healthcare professionals recognized the importance of youths’ development outside of the medical sphere.	Youths expressed distress due to the lack of consideration of their life and identity by healthcare professionals. Healthcare professionals described the difficult emotions that can accompany change.	Resource-person or social worker to support youths’ development and guide their pursuit of life projects.

## 3 Results

### 3.1 Participant information

A total of 54 interviews were conducted with 24 youths, 16 parents and 14 healthcare professionals. Nineteen participants were recruited in cardiology, 11 in nephrology, 10 in gastroenterology and 14 in adolescent medicine.

### 3.2 Perceptions of healthcare transition

Four main themes emerged when participants were asked about their perception of healthcare transition: (1) apprehension about adult care; (2) a lack of clarity about the transition process; (3) emotional attachment to paediatric healthcare professionals; (4) the significance of the coinciding transition into adulthood.

#### 3.2.1 Apprehension about adult care

Anticipating the transition evoked stress, anxiety, worry and apprehension amongst youths and their parents due to perceived differences between paediatric and adult care. The adult care system represented an unfamiliar and unknown environment, leading to worries about a loss of the high quality, personalized, thorough, and global care offered in the paediatric system, as these parents expressed:

“But I won’t hide from you that when you hear “you’re leaving Sainte-Justine it’s certain that you … how can I say it? You have prejudices like ‘my daughter will no longer be really protected’ in quotes. I don’t know if you understand, it’s like taking away … it’s the feeling of security that’s affected … ” (C-P1)

“It’s not that it’s worrying, it’s the anxiety, a bit … It seems to be really radical, the change, from Sainte-Justine to adult, the procedures, the way it’s done”. (C-P2)

#### 3.2.2 Lack of clarity about the transition

Lacking knowledge about the transition was a major source of stress for youths and parents who had not consistently received information on the details of their own transition ahead of time. This left parents and youths feeling “in the dark” about the future of their care, as this youth expressed:

“Pretty stressed out. I don’t have a lot of information about which hospital I will go to, which doctor, when, really, are we going to make the transition?” (C-Y1)

#### 3.2.3 Emotional attachment to pediatric healthcare professionals

The transition was particularly emotionally difficult for youths and their families as it meant a departure from a trusted paediatric care team with whom they had developed a close bond over numerous years of treatment. The difficulty of this change was also recognized by this paediatric healthcare professional:

“So, it’s certain that it’s a mourning that they have to do at the time of separation. So, it’s not negligible either, that separation, that mourning which comes with sadness, depending obviously on the relationship they had, which can vary from one child, one family to another, with the doctor, depending on the history they also knew with the environment … ” (G-HCP1)

#### 3.2.4 Transition into adulthood

Less commonly, though worth noting, the transition was perceived as an opportunity for change.

It was noted that the medical transition both coincides with, and signifies, a period of maturation into adulthood, best expressed by this youth:

“It’s always gradual, passing from childhood to adulthood, so for me, it’s really, it’s part of, one of the symbols of the passage. But now, this is more drastic. From, I mean from childhood to adulthood”. (C-Y2)

This period of change was often implied greater independence and new responsibilities for youths, as this youth described:

“Well, the transition, it’s to go from the state of my parents take care of pretty much every follow-up and me, I am, to I take over and I take this responsibility of managing the appointments and communicating with the center”. (G-Y1)

### 3.3 Salient concerns during healthcare transition

Table I presents 14 salient concerns related to healthcare transition identified by participants. These concerns are categorized in terms of content identifying and affirming the concern, content about the challenges related to this concern, and potential recommendations to address these concerns.

For example, on the concern for “continuity of care” (Table I), participants noted how they aspired to a smooth, fluid, and gradual process for which they would be prepared and in which they would be accompanied and guided. However, the reality is that youths and parents perceived the transition as an abrupt cut-off from care and, when applicable after transition, experienced a gap in care and lack of coordination and communication between paediatric and adult care (Table I). As result, a number of recommendations were made such as providing a transition coordinator to offer guidance throughout the transition and organizing pre-transition meetings with the patient, their parent(s), the paediatric care provider and the adult care provider doctor to facilitate communication between care centres (Table I).

Another salient concern was “Patient involvement in healthcare decisions”. Regarding this concern, participants affirmed the importance of youths’ participation in discussions and decisions surrounding their health and healthcare plan as well as the importance of making informed decisions in light of information on their medical condition and its implications (Table I). However, the reality also proved otherwise. Youths were not addressed or consulted on health-related issues, particularly in paediatric care. Some youths felt insecure making decisions without their parents and worried about making decisions alone in adult-centred care. Also, some parents were concerned about their child’s ability to make their own decisions (Table I). In response to these concerns, a number of recommendations were made such as that healthcare professionals promote active patient involvement in clinical practice., offer and explain choices to patients; and consult patients directly and listen to their perspectives on their care (Table I). Full details on the 14 salient concerns can be found in Table I

### 3.4 Flourishing and salient concerns regarding the transition

We sought to understand how different dimensions of flourishing—per Ryff and Singer’s (2008) integrative model of human flourishing (see online-only table: Key dimensions of human flourishing)—relate to important themes of the transition as identified by stakeholders. Dimensions of flourishing are also impacted by the 14 salient transition concerns identified (Table I).

#### 3.4.1 Environmental mastery

Though rarely if ever explicitly invoked literally by participants, environmental mastery proved to be a major theme recurring in the interviews, concerning the transition’s impact on youths’ sense of control, management, or security of their care. This dimension of flourishing was further elaborated on in terms of (1) the uncertainty due to the shifting care environments and (2) youths’ capabilities to assume agency in their care.

##### 3.4.1.1 Shifting care environments

The transition raised concerns about losing, sometimes abruptly, the high-quality care offered in the paediatric centre (See *Multidisciplinary care* and *Continuity in care*, Table I), as expressed by this parent:

“Because the patient is followed at Sainte-Justine, it’s been, let’s say, a few years, then afterwards, suddenly, we say “you’re no longer entitled to our services and you’re thrown into the adult world to fend for yourself”. (G-P1)

The precarity of an unfamiliar care environment emerged as a significant source of worry (See *Adapting to a new care system*, Table I). Hence, participants valued greater information and communication about the transition (See *Knowledge and information about the transition*, Table I):

“It’s reassuring to know where we’re going, what needs to be done.” (N-P1)

Overall, more information prior to the transition, and more information about the adult healthcare team were desired (Table I). For instance, some youths and their families lacked information about the timing of the transition, the care centre and care team to which they would transfer, and their role in coordinating the transfer of care. They suggested that pamphlets or informational videos on the transition process and group information sessions would help them feel better prepared. Another recommendation was to produce an informational guide about the new care centre, the adult healthcare system, and references to relevant resources (Table I).

##### 3.4.1.2 Agency in care

Insecurity and anxiety arose when youths had not developed the capabilities to manage and advocate for their health, responsibilities foregrounded by parents throughout paediatric care (See *Parental involvement in healthcare; Health management; Patient involvement in healthcare decisions*, Table I):

“ … having to manage my appointments. Well, dealing with my prescriptions, my medications and all that. It makes me think a little bit more about that. Because now, it’s my parents that do it. So, well, this, it’s something that I don’t have to do, but that I will have to do. From this side of things, it sure does stress me a bit”. (C-Y4)

Greater medical knowledge proved primordial to youths’ active participation in their care (See *Knowledge and information about the medical condition*, Table I) and it was recommended that paediatric healthcare professionals provide an in-depth explanation of youths’ medical condition (e.g., their diagnosis, treatment history, illness manifestations) and evaluate patients’ knowledge before the transition. In this regard, references to websites containing information on the youth’s medical condition or more specifically, a notebook or pamphlet recording the details of the patient’s medical condition and history could prove useful.

#### 3.4.2 Autonomy

Autonomy emerged as a central dimension of the transition that was widely understood in terms of youths’ functional independence in concrete self-care tasks (See *Health management*, Table I):

“Autonomy? Well, it’s making your appointments by yourself, everything, by yourself, well really doing everything by yourself … So, if you know that you have an appointment soon, you know, you mark it down somewhere so you don’t forget about it. That, I think is a part of autonomy. Or if you think you’re starting to have health problems, making an appointment on your own with your doctor”. (G-Y2)

However, stances on autonomy varied widely as independence was neither universally desired nor attainable (See *Parental involvement in healthcare; Variability in autonomy*, Table I), notably because some youths are not psychologically ready at the time of transition to assume all decisions and be autonomous agents with respect to their care or that this goal is not compatible with the condition of the youth. Further, for youths and parents alike, autonomy was not always valued over the support of parental accompaniment which can help ease stress and improve communication in medical consultations. Contrasting perspectives on autonomy were reflected across the interviews:

“I would tell you that there’s a good 60% of our young adults, at the time we transfer them, who are still very dependent on their parents”. (N-HCP1)

Youths also anticipated greater autonomy in the governance of their care by voicing their perspectives on health-related issues (See *Patient involvement in healthcare decisions*, Table I). However, participants had diverging opinions on youths’ ability to make their own decisions, as this parent expressed:

“But it’s true that at this point, at 18 years old, it’s [hidden name] who will decide kind of what happens. I find that difficult nevertheless. It’s true, I find it difficult to say that it’s him that will have the last word on everything … ” (N-P1)

Youths’ agency in their care would require greater respect on the part of healthcare professionals, which was sometimes undermined in paediatric care:

“So I think that the treatment team has to be open to the fact that the patient, he has knowledge, you know, like, more in-depth knowledge about his illness”. (G-P2)

Respect for confidentiality elicited further concerns about youths’ independence in healthcare consultations and autonomy in decision-making, notably on behalf of worried parents (See *Confidentiality*, Table I).

#### 3.4.3 Positive relations with others

Positive relations with others covered the important relationships constituting the interpersonal network of youths with chronic illnesses, namely, the importance of familial and parental support, trust in healthcare professionals, and connection with peers.

##### 3.4.3.1 Relations with family and parents

Parents offered affective support and reassurance aligned with the desire for ongoing parental accompaniment into adult care (See *Parental involvement in healthcare*, Table I):

“I’m touched by this, I’m lucky. My daughters trust me and they want me there, but I’m really comforted, so many things happen I’m happy to be there. The longer we can do things for our children, the better it is”. (AM-P1)

Youths similarly valued the comfort and security of their parents’ presence:

“I would like my parents to come … I get enormously stressed when I go to the hospital about whether it’s going to be good news this time, or some bad news”. (C-Y1)

Related to these tight relationships with parents, concerns related to respect for confidentiality (See *Confidentiality*, Table I) reflected the difficulties of parents’ exclusion from youths’ care:

“And then as I said, frequently, it’s the parent/children separation, that is maybe difficult. Not that it’s not desirable, either for the children, or for the parents. Sometimes, maybe it’s easier for one side than the other”. (C-HCP1)

##### 3.4.3.2 Relations with healthcare professionals

Leaving their paediatric care team, with whom they had formed close ties, was a major source of stress for youths and parents (See *Relational continuity with healthcare professionals*, Table I). Due to this privileged bond, they worried about establishing trust in new healthcare professionals:

“ … doctors that we have they know us since I was born. We trust them, and they do a really good job … And then it’s one person who, in fact, later he will know us even more, but it won’t ever be exactly as much as … he won’t have seen us grow as much”. (N-Y1)

##### 3.4.3.3 Relations with peers

Youths could feel set apart socially due to their chronic illness (See *Social connection*, Table I). They desired greater understanding and awareness of their medical condition from others and valued opportunities to identify with other youths living with chronic illness “who are living the same thing as you”. (G-Y1)

#### 3.4.4 Self-acceptance

Self-acceptance was largely understood in terms of youths’ acceptance of the implications of their chronic illness. These issues did not bear an explicit link to the medical transition but were considered important for the flourishing of youths.

Youths face discouragement, frustration and defeat when unable to engage in certain activities, notably physical activities, due to the daily implications of their medical condition. (See *Navigating life with chronic illness*, Table I). Facing and accepting their limits was considered necessary to the development of a positive outlook on their self and life.

The need to identify with others proved central to youths’ self-acceptance (See *Social connection*, Table I). Youths related self-acceptance to a sense of normality and belonging with others and struggled with feeling set apart due to their chronic illness:

“People, they don’t understand. Let’s say I’m taking a medication that makes it impossible for me to drink, and then ‘Come on! One drink?’ And then I go no, I can’t. That, it’s just an example. And I know very well that in other areas, there are other cases where it’s the same thing. It’s all the same, but this is an example of, here, it makes you feel a little bad. That’s what it is”. (G-Y1)

The opportunity to connect and exchange with other youths with chronic illnesses was recommended as a means of affirming that they were not alone in their experience.

“Let’s say you know someone else your age living the same thing, I don’t know, you can like understand more and put yourself in the other person’s shoes more and better understand because you’re living the same thing, you’re the same age”. (G-Y3)

#### 3.4.5 Purpose in life

Purpose in life was one of the dimensions of human flourishing less frequently discussed amongst participants. This dimension related to the transition of care insofar as the transition coincides with a period wherein youth are discovering and navigating their life path and interests, a crucial moment in developing a sense of purpose.

Youths’ sense of direction in life was namely characterized by engagement in recreational passions and the pursuit of academic and career objectives. Youths faced notable challenges in their pursuit of life objectives due to the limitations posed by their chronic illness (See *Navigating life chronic illness*, Table I). For example, this parent recounts explaining their child’s limitations:

“Well, choices on sports, yes, we made a choice. We had to explain it to him. He plays hockey. We had to stop him because it was too dangerous because of contact, you know. And, yes, he loves hockey. He would have liked to play, but he understands the situation. We explained it to him well, you know, why if there is an impact what could happen. And that’s it”. (C-P3)

Youths expressed the desire for greater consideration of their life projects aspirations from healthcare professionals who could provide guidance on youths’ life path in light of their medical condition:

“ … my daughter, she already has a vision of where, what’s the career that she wants, what domain she wants to be in. It’s just, but just to not really guide her, but just to see that if it’s … just to have a discussion to just, to say ‘Ok, yes this is realistic or not’.” (G-P1)

#### 3.4.6 Personal growth

Personal growth was one of the dimensions of human flourishing minimally discussed in interviews. Nevertheless, the content of these responses provided a substantial vision of the factors that characterized the passage from adolescence into adulthood. The transition represented, and coincided with, this broader life transition:

“ … it’s like symbolic, it’s really … because it’s rare, you know. It’s always progressive, passing from childhood to adulthood, so for me, it’s really, it’s a part of, one of the symbols of the passage. But, this is more drastic. From the life, I mean from childhood to adulthood. This it’s really, I have the impression of being like Mowgli when he becomes an adult, no but [laughs]! No, but it’s really, it’s really symbolic … ”” (C-Y2)

Participants drew attention to the important aspects of youths’ development in life spheres beyond the medical sphere (See *Social connection* and *Navigating life with chronic illness*, Table I). This broader vision of development was characterized by socialization with peers; gaining independence; engagement in work, education and recreational activities; and setting objectives for the future. These aspects contributed to “a normal life” (G-Y3) or the growth (N-HCP2) of youths.

Youths’ growth was fostered through their pursuit of academic, professional, and recreational objectives which emerged as a distinctive concern given the particular impact of chronic illness on engagement in these spheres (See *Navigating life with chronic illness*, Table I).

## 4 Discussion

Healthcare transition is a complex process that poses several challenges for youths’ medical care and coincides with a period of development wherein youth are navigating through changes in different dimensions of their life. Few studies have explored what transition means and implies in terms of a flourishing life for youths (Lanteigne et al., 2021). Further, the perspectives of youths and parents are currently underrepresented in research on transition (Okumura et al., 2022). This study set out to investigate the perspectives of youths, parents, and healthcare professionals on healthcare transition with an orientation to youths’ pursuit of a flourishing life to broaden the perspective on what transition is and means for youths.

Many participants reported that difficulties encountered during transition were related to important dimensions of flourishing. Parental involvement in healthcare seemed to be a central concern pertaining to flourishing as it touched on three dimensions of flourishing (environmental mastery, autonomy, positive relation with others). This is understandable as youths with chronic conditions and their parents often have a unique relationship fostered by the many years of caring for their medical condition as noted in previous research (Larivière-Bastien et al., 2013). Approaching adulthood, parents’ role in the management of their youths’ health is expected to change, which can create tensions and jeopardize positive relationships between them. Although most of the concerns were closely linked with environmental mastery and autonomy, some were also related to personal growth, self-acceptance, and purpose in life such as “social connection”, “navigating life pursuits with a chronic illness” and “holistic development” (see Table I). Though these dimensions (personal growth, self-acceptance, purpose in life) of flourishing were less recurrent in the data, they are significant to the lives of youths with chronic illness. In our discussion we focus on: (1) the transition’s impact on the basic need for security of care in terms of environmental mastery; (2) the contextual and relational ways in which youths’ autonomy is envisioned; (3) the holistic and humanistic aspects of transition.

### 4.1 Environmental mastery and the unsettling nature of the transition

Our study revealed that the transition poses crucial challenges for youths’ sense of “mastery” due to the marked change of the context in which they will receive care. This was very clearly expressed in the widespread worries about the continuity of their care. These worries and the general apprehension towards the transition observed are exacerbated by perceived disparities between adult and paediatric health professionals and the general lack of information about transition. Such concerns are congruent with previous findings on the transition of care in Quebec (Kakkar et al., 2016; Nakhla et al., 2017; Oskoui, 2012) as well as similar international findings (Gray et al., 2017). These new findings show the imminence and extent of challenges pertaining to environmental mastery as a threat to youths’ general well-being in the transition.

The implications of these experiences are explainable by the impact of the lack of control or perceived lack thereof and lower environmental mastery on experiencing stress more generally (Montpetit & Tiberio, 2016; Neupert et al., 2007). Where environmental mastery in part designates the ability to choose and shape suitable environments where one fits and thrives, the often-unsought change from a trusted care environment can undermine one’s sense of control of the situation and generate stress. It is indeed very difficult to feel efficacious about a situation which is poorly understood and worrisome. Accordingly, our findings support measures (e.g., structured coordination of the transition, preparatory education about the transition and the youths’ medical condition, sharing of experiences between youths) that will reduce stress by promoting greater efficacy and mastery of one’s context. Several relevant practices have been proposed to provide information and guidance (e.g., transition coordinators (Annunziato et al., 2013; Gray et al., 2018) and joint transition clinics (Crowley et al., 2011). It would be crucial to understand how such strategies can impact the sense of efficacy and mastery of youths and their parents. However, while offering structured support can be valuable to foster security in a period of change, it may not directly empower youths. For instance, the top-down implementation of structured transition programmes and delivery of transition information could disempower youths by overriding their ability to advocate for their needs in the transition and interfere with the development of solutions to the obstacles they face. This could impede environmental mastery if the proposed solution undermines the sense of control over the situation.

### 4.2 Autonomy and the contextual and relational aspects of care

Our findings show that youths’ autonomy was commonly equated to their functional independence. Autonomy as independence is pervasively privileged as a primary objective of healthcare transition (Hamdani et al., 2015; Lanteigne et al., 2021). It is well reflected in common transition tools that evaluates youths’ self-management capabilities such as the TRAQ (Wood et al., 2014), the TRANSITION-Q (Klassen et al., 2015) and the Montreal Children’s Hospital transition readiness assessment (Montreal Children’s Hospital, 2023). However, this focus of transition care has been challenged from an ethical and psychosocial standpoint because not all youths value autonomy as a form of independence (Hamdani et al., 2015). For example, a previous study which gave the opportunity to youths with cerebral palsy to express their concerns about transition care found that they mostly envisioned autonomy as a relational ability rather than a form of individualism or independence from their parents (Larivière-Bastien et al., 2013). Our findings do show that the development of self-management skills and knowledge is commonly accepted by youths and parents as an important objective of the transition. However, the emphasis on youths’ independence from their parents does not reflect the values, needs, or realities of all youths and their parents.

Our results, and those of previous studies, evidence the need to move away from individualistic accounts of autonomy towards a contextualized account of autonomy. A contextualized account of autonomy acknowledges its importance as a key dimension of flourishing while recognizing the influence of agential and contextual factors as well as accommodating for the varying autonomous capacities of youths (Bogossian et al., 2020; Lanteigne et al., 2021). It recognizes that autonomy is not an absolute good but exists in a broader framework of values (Larivière-Bastien et al., 2013). This also corresponds to youths’ values of support and security which were not outweighed by autonomy. Whereas parental involvement is typically posited as an obstacle to youths’ autonomy (Acuña Mora et al., 2020; Stewart et al., 2017; While et al., 2017), our results reflect the existence of mutual agentivity and dependency in the transition (Carnevale et al., 2017). The contextualized account of autonomy helps explain the needs and objectives of youths by nurturing the development of health management skills and knowledge, thus favouring a more gradual transfer of responsibility and growth towards autonomy. Accordingly, sensitization to the contextual and relational aspects of autonomy (Larivière-Bastien et al., 2013) is of particular importance for the transition. Youths’ autonomy may be augmented by developing agency in their care, rather than meeting the expectations of others. Increasing youths' health literacy, i.e., knowledge of their chronic illness, is a crucial contribution to promoting their sense of self-efficacy. Moreover, healthcare professionals should address youths directly on issues surrounding their health, recognizing youths as self-experts and self-advocates, to empower and promote their agency in their care (Miller et al., 2018).

### 4.3 Holistic and humanistic aspects of the transition

Assessing the transition with an orientation towards youths’ flourishing permitted the consideration of the broader life aspirations and interests of youths, a topic infrequently tackled in transition care which tends to be more narrowly oriented towards traditional clinical outcomes such as medical outcomes, and even more narrowly medication and treatment compliance (Lanteigne et al., 2021). While it has been long recognized that transition care should integrate support for youths’ vocational, recreational and educational aspirations (Blum et al., 1993; Toulany et al., 2022), these aspects are still poorly integrated in existing transition programmes (Lanteigne et al., 2021).

Our results show that youths desired healthcare professionals to consider them as individuals with values, interests, and aspirations that are equally at stake in their medical treatment. Though the possibility of personalized healthcare is sometimes limited, these perspectives call for a previously recommended progressive paradigmatic shift in attitudes (Racine et al., 2014) wherein the interests and values of youths as persons are put at the centre of the design, development, implementation, and evaluation of healthcare. Some existing transition interventions, such as the ON TRAC model (Minskoff & Demoss, 1993; Paone et al., 2006) and the Youth KIT (CanChild, 2023; Freeman et al., 2015; Gorter et al., 2015) integrate educational, vocational and recreational objectives and are worth citing. However, these resources tend to be oriented towards planning for the future (e.g., professional activities), which does not fully respond to the desires of youths for information on educational, vocational, and recreational activities found in this study. The recommendations received in our interviews ascertain the need for greater integration of such guidance in clinical practice (Gorter et al., 2015). Granting more attention to youths’ life aspirations could increase self-esteem and facilitate positive peer relationships (Lee et al., 2019). The opportunity to connect with other youths with chronic illness in peer support groups helps reduce the sense of social isolation and improve self-acceptance and encourage a sense of normalcy (Olsson et al., 2005). In this light, participatory models of transition that engage patient and parent perspectives in care development are promising routes to empower the human and holistic aspects of persons during the transition (Willen, 2022).

### 4.4 Strengths & limitations

We recognize several limitations of this study. Our population was limited to one paediatric care centre in Montreal, Quebec and mostly focused on physical conditions, although the challenges identified could be relevant to mental (Anderson et al., 2022) and neurodevelopmental conditions (Racine et al., 2014; Swift et al., 2013). Adolescent medicine (one of the four clinics included in this study) includes a significant number of patients with psychiatric conditions or psychiatric comorbidities, though the clinic of adolescent medicine covers not only or strictly mental healthcare. This limits the generalizability of these perspectives on the transition, but the qualitative design is intended to favour contextual relevance and connection. In fact, pragmatically oriented transition research does not aim towards such rigid generalizability because transition interventions depend on a multitude of contextual factors (e.g., clinical, geographic) and should respond to the specific needs of the users of these services (Frega & Carreira da Silva, 2011). Certain limits also exist with respect to the application of a theoretical framework to interpret lived experiences. The dimensions of well-being, as defined in the theoretical framework of human flourishing (Ryff & Singer, 2008), may not translate to colloquial understandings of these concepts. In interviews, more common concepts such as “autonomy” may be discussed more by participants than less familiar ones such as “environmental mastery”. This required researchers to occasionally extrapolate from participants’ perspectives to substantiate these dimensions. Further, this required a certain degree of adaptation of the theoretical definitions of these dimensions which did not reflect explicitly the contextually situated and lived experiences of participants. This would warrant further dedicated investigation of human flourishing in this context, especially given the limited research on flourishing in youths (Witten et al., 2019). Despite its limitations, this framework proved to be a fruitful strategy to contribute to a better understanding of what matters for youths during transition.

## 5 Conclusion

The healthcare transition for youths with chronic condition coincides with a period during which they experience several other life changes, amounting to a highly stressful and impactful period of life. In response, transition care programmes have received greater attention and developed substantially in recent years. Yet these programmes marginally engage with deeper considerations related to human flourishing and the objectives and practices embedded in transition care may not respond to the aspirations of youths. This study is one of the first to advance in this direction by explicitly drawing upon the literature on human flourishing and stakeholder experiences to investigate the broader personal and social context of transition care. The notable impact on youths’ sense of environmental mastery and autonomy reinstates the profound human impact of the transition and transition interventions. Meanwhile the existential aspects of the transition call for greater consideration of the relational dimensions of youths’ care. In subsequent steps of this ongoing research project, we plan to engage with youths, parents, and healthcare professionals on important dimensions of human flourishing through participatory methods. Based on the findings from the interviews and the survey, we have now embarked on the design of two participatory interventions: (1) a series of informational and awareness-raising short videos targeting youth approaching the transition and (2) interactive awareness-raising dialogues with relevant clinical teams. We hope to be able to report on these steps in forthcoming publications.

## Supplementary Material

Supplemental Material

## Notes

1. In Ryff and Singer’s model, the concept of flourishing and the concept of psychological well-being are used interchangeably. We only use human flourishing given its deep theoretical connection to ethics and also avoid using two terms.

2. The COVID-19 pandemic undermined our ability to truly instil a strong participatory orientation during the initial phase of the study reported here. We had to change our approved protocol to move from focus group interviews to individual interviews and the entire spirit of engagement and participation we were seeking was unfortunately compromised. We are now pacing ahead with a participatory orientation. So, despite our intents, this study is a qualitative study that serves as a basis for further participatory research.
