# Supplementary material for: Transition from Paediatric to adult health services: Aspirations and practices of human flourishing
Source: Int J Qual Stud Health Well-being. 2023 Nov 23;18(1):2278904. doi: 10.1080/17482631.2023.2278904 (PMC11000676; doi:10.1080/17482631.2023.2278904)
Supplement: Supplemental Material [file ZQHW_A_2278904_SM6840.docx]

**Online-only table: Key dimensions of human flourishing**

| **Environmental mastery** |
| --- |
| Environmental Mastery concerns the ability to “choose or create contexts suitable to personal needs and values”; “the capacity to act on and change surrounding world through mental and physical activities; and gaining a “sense of control over [the] external world” (Ryff & Singer, 2008). Environmental mastery reflects an individual’s sense of control in their life (Montpetit & Tiberio, 2016) and designates an individual’s perception of their ability to manage life situations (Gao & McLellan, 2018). |
| **Autonomy** |
| An autonomous person is “self-determining and independent” and “regulates behavior from within” (Ryff & Singer, 2008). Autonomy, classically understood in terms of self-governance, manifests in actions that are deemed intentional, informed by a degree of understanding, and are not under the constraint of controlling influences (Beauchamp & Childress, 2001; Beauchamp & Childress, 2009). Individualistic conceptions of autonomy have been critiqued for narrow constructions of the self as an independent and rational agent. These concepts have given way to relational (Mackenzie & Stoljar, 2000) and contextualized (Racine et al., 2021) accounts of autonomy, including in the context of transition care (Bogossian et al., 2020; Racine et al., 2014; Racine et al., 2013). |
| **Positive relationships with others** |
| This dimension of well-being designates …. “The interpersonal realm as a central feature of a well-lived life”; “strong feelings of empathy and affection”; “close, trusting relationships”; and “close identification with others” (Ryff & Singer, 2008). Positive relations with others are considered fundamental to human development and well-being (Roffey, 2012). They offer a sense of connection, support, and belonging by feeling valued by another person and have shown to support resilience in the face of adversity (Roffey, 2012). |
| **Self-acceptance** |
| Self-acceptance pertains to… a “positive self-regard” in light of acknowledgement and acceptance of “multiple aspects of self, including good and bad qualities” (Ryff & Singer, 2008). It is a central element to a person’s psychological and emotional well-being (MacInnes, 2006). Self-acceptance can be undermined by critical self-evaluation, self-evaluations based on others’ perceptions, and comparison to others (Carson & Langer, 2006) and is contrasted with self-depreciation (Bernard et al., 2013). Positive self-acceptance is characterized by a sense of self-worth in light of awareness and acceptance of one’s weaknesses or flaws (Bernard et al., 2013). |
| **Purpose in life** |
| Purpose in life pertains to… “A sense of directedness”; “Aims and objectives for living”; “purposes or goals that characterize different life stages” (Ryff & Singer, 2008). It reflects a general positive orientation that a person regarding their life trajectory as well as an orientation toward what are meaningful experiences and endeavors to them. Having purpose in life reflects a state and orientation exhibiting that one’s life course is aligned with important values and intrinsic motivations. It is an important aspect of psychological and even physical well-being (Alimujiang et al., 2019), although there are varying findings on this point (Almeida et al., 2020; Robitschek, 1998). |
| **Personal growth** |
| Personal Growth is defined by “a continual process of developing one’s potential” …“continued development”… “openness to experience”…”confronting of new challenges at different periods of life”…”Realization of personal potential” (Ryff & Singer, 2008). Personal growth can be stimulated by developmental processes, environmental changes, or the individual’s own initiative (Robitschek, 1998). The capacity for growth supports individuals’ ability to adapt to life changes (Robitschek, 1998). |

**Table references**

Alimujiang, A., Wiensch, A., Boss, J., Fleischer, N. L., Mondul, A. M., McLean, K., Mukherjee, B., & Pearce, C. L. (2019). Association between life purpose and mortality among US adults older than 50 years. *JAMA Network Open*, *2*(5), e194270. doi:10.1001/jamanetworkopen.2019.4270

Almeida, V. M., Carvalho, C., & Pereira, M. G. (2020). The contribution of purpose in life to psychological morbidity and quality of life in chronic pain patients. *Psychology, Health & Medicine*, *25*(2), 160-170. https://doi.org/10.1080/13548506.2019.1665189

Beauchamp, T., & Childress, J. (2009). *Principles of biomedical ethics* (6th ed., Vol. ). Oxford University Press.

Bernard, M. E., Vernon, A., Terjesen, M., & Kurasaki, R. (2013). Self-acceptance in the education and counseling of young people. In Bernard, M. (Eds.), *The strength of self-acceptance* (pp. 155-192). Springer. https://doi.org/10.1007/978-1-4614-6806-6_10

Bogossian, A., Majnemer, A., & Racine, E. (2020). Contextualized autonomy in transitional care for youth with neurologic conditions: the role of the pediatric neurologist. *Journal of Child Neurology*, *35*(8), 536-542. https://doi.org/10.1177/0883073820918454

Carson, S. H., & Langer, E. J. (2006). Mindfulness and self-acceptance. *Journal of Rational-Emotive and Cognitive-Behavior Therapy*, *24*(1), 29-43. https://doi.org/10.1007/s10942-006-0022-5

Gao, J., & McLellan, R. (2018). Using Ryff’s scales of psychological well-being in adolescents in mainland China. *BMC Psychology*, *6*, Article 7. https://doi.org/10.1186/s40359-018-0231-6

MacInnes, D. L. (2006). Self‐esteem and self‐acceptance: an examination into their relationship and their effect on psychological health. *Journal of Psychiatric and Mental Health Nursing*, *13*(5), 483-489. https://doi.org/10.1111/j.1365-2850.2006.00959.x

Mackenzie, C., & Stoljar, N. (2000). Introduction: autonomy refigured. In Mackenzie, C., & Stoljar, N. (Eds.), *Relational autonomy: feminist perspectives on autonomy, agency, and the social self* (pp. 3-31). Oxford University Press.

Montpetit, M. A., & Tiberio, S. S. (2016). Probing resilience. *The International Journal of Aging and Human Development*, *83*(4), 311-332. https://doi.org/10.1177/0091415016655162

Racine, E., Bell, E., Yan, A., Andrew, G., Bell, L. E., Clarke, M., Dubljevic, V., Goldowitz, D., Janvier, A., McLachlan, K., Muhajarine, N., Nicholas, D., Oskoui, M., Rasmussen, C., Rasmussen, L.A., Roberts, W., Shevell, M., Wade, L., & Yager, J. Y. (2014). Ethics challenges of transition from paediatric to adult health care services for young adults with neurodevelopmental disabilities. *Paediatrics & Child Health*, *19*(2), 65-68. https://doi.org/10.1093/pch/19.2.65

Racine, E., Kusch, S., Cascio, M. A., & Bogossian, A. (2021). Making autonomy an instrument: a pragmatist account of contextualized autonomy. *Humanities and Social Sciences Communications*, *8*(1), 139. https://doi.org/10.1057/s41599-021-00811-z

Racine, E., Lariviere-Bastien, D., Bell, E., Majnemer, A., & Shevell, M. (2013). Respect for autonomy in the healthcare context: observations from a qualitative study of young adults with cerebral palsy. *Child: Care, Health and Development*, *39*(6), 873-879. https://doi.org/10.1111/cch.12018

Robitschek, C. (1998). Personal growth initiative: the construct and its measure. *Measurement and Evaluation in Counseling and Development*, *30*(4), 183-198. https://doi.org/10.1080/07481756.1998.12068941

Roffey, S. (2012). Introduction to positive relationships: evidence-based practice across the world. In Roffey, S. (Eds.), *Positive Relationships* (pp. 1-15). Springer. https://doi.org/10.1007/978-94-007-2147-0_1

Ryff, C. D., & Singer, B. H. (2008). Know thyself and become what you are: A eudaimonic approach to psychological well-being. *Journal of Happiness Studies*, *9*, 13-39. https://doi.org/10.1007/s10902-006-9019-0
